# Stressing about misplaced fat is a key to longevity

**DOI:** 10.7554/eLife.10161

**Published:** 2015-08-19

**Authors:** George A Lemieux, Kaveh Ashrafi

**Affiliations:** Department of Physiology, University of California, San Francisco, United States; Department of Physiology, University of California, San Francisco, United Stateskaveh.ashrafi@ucsf.edu

**Keywords:** aging, fatty acid signaling, germline stem cells, lipid metabolism, proteostasis, SKN-1/Nrf, *C. elegans*

## Abstract

The abnormal accumulation of fat increases the lifespans of nematodes that lack sex cells.

**Related research article** Steinbaugh MJ, Narasimhan SD, Robida-Stubbs S, Moronetti Mazzeo LE, Dreyfuss JM, Hourihan JM, Raghavan P, Operaña TN, Esmaillie R, Blackwell TK. 2015. Lipid-mediated regulation of SKN-1/Nrf in response to germ cell absence. *eLife* **4**:e07836. doi: 10.7554/eLife.07836

**Image** Some mutant worms live longer than wildtype worms

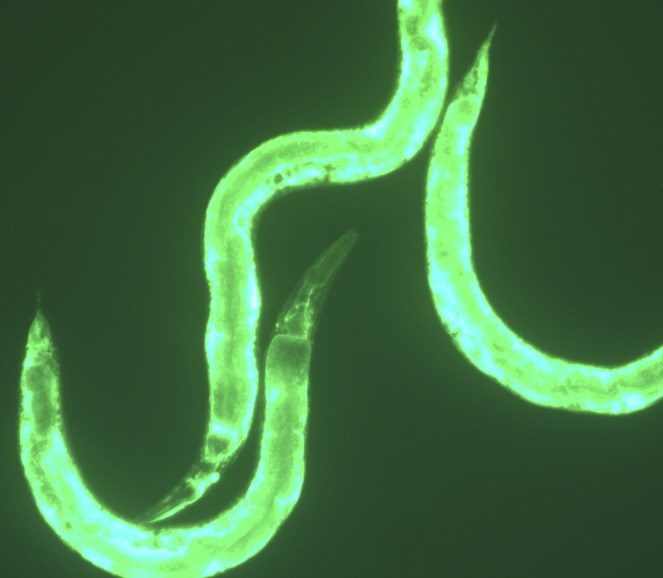

Germ line tissue – the tissue that produces egg and sperm cells – is known to influence the lifespans of several animal species (Kenyon, 2010). For example, removing this tissue from the gonads of the worm *Caenorhabditis elegans* extends the lifespans of hermaphrodite individuals by about 60%. However, this effect is not due to the loss of fertility because the removal of the entire gonad, which includes both the germ line and the ‘somatic’ gonad, does not alter the worm's lifespan (Hsin and Kenyon, 1999).

The prevailing interpretation of these findings is that the gonad must produce signals that increase or reduce lifespan. As such, the hunt has been on to identify these ‘aging’ signals. Now, in *eLife*, Keith Blackwell and colleagues at the Joslin Diabetes Centre – including Michael Steinbaugh as first author – report that the increase in lifespan is the consequence of the stress response that is initiated upon the abnormal accumulation of lipids after the germ line cells are removed (Steinbaugh et al., 2015).

This work was performed on worms that lacked the stem cells that become eggs and sperm cells (Hubbard, 2007). In addition to living longer than normal worms, these *glp-1* mutants exhibit elevated levels of stress resistance and other processes associated with increases in lifespan in other animals. They also have higher levels of lipids, leading to the idea that the extended lifespan is due to the accumulation of beneficial lipids (Ackerman and Gems, 2012; Lapierre and Hansen, 2012).

Steinbaugh et al. investigated the role of SKN-1 – a transcription factor that is involved in stress resistance, metabolism and lifespan extension in many animal species – in *glp-1* mutant worms. They found that a deficiency in germ line cells leads to the activation of SKN-1 and that this activity is required for the increase in lifespan. By profiling the changes in gene expression caused by germ line deficiency, they demonstrated that SKN-1 influences the expression of genes that have roles in stress resistance, protein homeostasis and lipid metabolism.

The study also shows that, in *glp-1* mutants, an unsaturated fatty acid called oleic acid, or a related metabolite, acts as a signal that leads to the activation of SKN-1. Similarly, a metabolite of oleic acid was recently shown to promote the activities of two other transcription factors called NHR-80 and NHR-49 (Folick et al., 2015). These transcription factors are also required for the extended lifespan of *glp-1* mutants (Goudeau et al., 2011; Ratnappan et al., 2014).

So, are these lipids elixirs of longevity? A provocative answer provided by Steinbaugh et al. is that *glp-1* mutants are fat because they accumulate excessive quantities of yolk, which transports nutrients from the intestine to the egg cells in the germ line tissue. Yolk particles – which are made of lipids and proteins – are produced in intestinal cells, secreted and then taken up by the developing egg cells (Grant and Hirsh, 1999; Figure 1). As an individual *C. elegans* hermaphrodite is capable of producing its body weight in progeny every day, any changes to the flow of yolk particles to the germ line tissue can dominate the overall fat content of the organism. In the *glp-1* mutants, the intestinal cells continue to produce copious amounts of yolk, which accumulates due to the lack of germ line cells (Figure 1).

**Figure 1.**
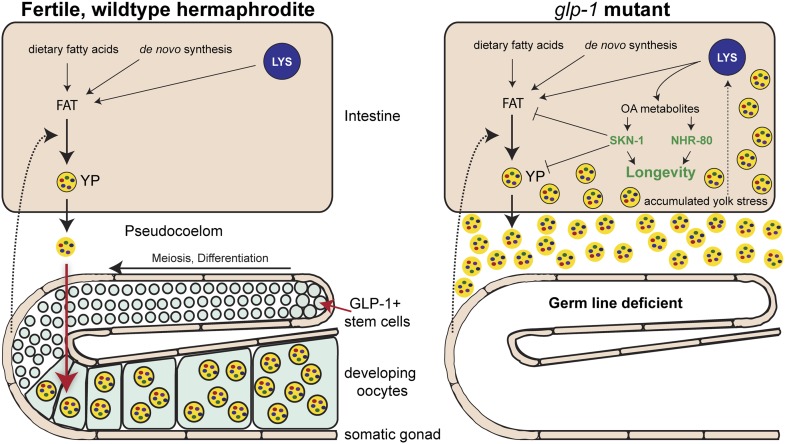
How the accumulation of fats leads to increased longevity in mutant worms. Intestinal fat is derived from different sources including dietary fatty acids, new (de novo) synthesis and cell compartments called lysosomes (LYS). In wildtype hermaphrodite worms (left), lipids are packaged into yolk particles (YP), which are then secreted into a cavity called the pseudocoelom and taken up by egg cells (oocytes) in the germ line. The signals that promote yolk production have not yet been identified, but are likely to come from cells in the somatic gonad (dotted arrow). In the *glp-1* mutants (right), the stem cells that give rise to the egg cells are absent: however, these sterile worms still produce yolk particles, which accumulate in the body and trigger a stress response. This response involves lipase enzymes (not shown) within the lysosomes that produce metabolites of oleic acid (OA). These molecules act as signaling molecules to activate, either directly or indirectly, the transcription factors NHR-80 and SKN-1, which promote longevity. One of the roles of SKN-1 is to limit the accumulation of fat and yolk.

Significantly, Steinbaugh et al. showed that animals lacking germ line cells and SKN-1 accumulate even more fat, consistent with this protein having a role in upregulating the mechanisms that burn fat. As such, the activation of SKN-1 in animals lacking germ line cells helps to reduce the burden caused by the excess lipids and the resulting accumulation of yolk (Figure 1). In the process of dealing with the stress of elevated lipid levels, animals turn on molecular mechanisms that extend lifespan.

These findings indicate that the longevity of *glp-1* animals is not a simple consequence of the accumulation of beneficial fats; rather, it is provoked by the inappropriate distribution of yolk in tissues. However, this study, does not rule out the possibility that the gonad sends out additional signals that influence lifespan.

By revealing that the excess lipids in *glp-1* mutants are likely due to the accumulation of yolk, the work of Steinbaugh et al. starts to address another conundrum: many of the changes in gene expression that are activated in the *glp-1* mutants (and are required for the longevity of the mutants) can also be activated by nutrient deprivation in wildtype worms. This suggests that while the yolk and stored lipids are chemically and biologically related, they have different functions and should not be treated as a single entity, as most studies of *C. elegans* fat content currently do. Finally, given the similarities between the *C. elegans* intestine and the mammalian liver, the results of this study also suggest that the mammalian equivalents of SKN-1, the Nrf transcription factors, may limit susceptibility to non-alcoholic fatty liver disease in a similar way.
